# The Hygiene and Advantage of Illness

**Published:** 1893-09-23

**Authors:** 


### The Hygiene and Advantage of Illness.

Sickness has been treated under many aspects, and it possesses an interest all its own. We propose to deal with it from the hygienic and moral standpoint. It is natural for the hale man or woman to regard illness with horror. Many fortunate people live so many years without ever being really ill that their sympathies with suffering become deadened, and may even cease to exist. We well remember an experience of this kind, which will come home, no doubt, to some readers. For many years it was our lot to spend the greater portion of each day in a hospital, surrounded by disease and suffering of all kinds. Providence mercifully ordains that those who have to be constantly with sick people grow to regard them from the point of view of how best to promote their comfort and recovery. This becomes an absorbing interest, and so the habitual attendant gradually loses all feeling of repulsion and dread. A casual visitor to a hospital ward is appalled by the evidences of suffering and sickness which surround him. Cases are recorded, and have indeed happened within our experience, where visitors cannot bring themselves to remain in a ward, and have perforce to leave it, or they suffer from collapse. It must not, however, be supposed, that the casual visitor has necessarily more sympathy with suffering than the habitual attendant. Indeed, it may be quite the contrary, the two effects being accounted for by the fact that the attendant is under self-discipline, whilst the visitor is not.

In our own case this discipline and the volume of work which had to be regularly mastered in the hospital, pushed the sufferings of the patients into the background. We were unaware of the fact at the time, but a severe attack of illness forced it upon our attention. As we lay in the hospital suffering and helpless we began to realise something of the measure of the misery of the many thousands, who had found it a house of recovery. This led to a keener appreciation of the privileges of being permitted to minister to the needs of helpless and suffering humanity. Our own illness thus became an education in itself, and led to results so beneficial as to fill the heart with gratitude and joy. Here then is one aspect of illness which all should strive to realize and profit by. Martial has truly said that life is not to live but to be well. We are, however, convinced, that there is after all a privilege in being ill, which the most hale and robust may well envy the sick. Experience is a hard master, but his lessons possess a value more than commensurate with the pains he inflicts. Thus illness may make a hard man tender, a violent man patient, and a selfish man considerate for the feelings of others, for the rest of his life.

We have thus far dealt with some of the moral aspects of sickness. What then of its hygiene? It may be argued that illness cannot be hygienic—is this so? Who is there that has watched a splendid yacht travelling at great speed under full sail, and noted the moment when the squall struck it with such violence that it lay crippled on the face of the waters, and has not felt a pang of poignant grief? In the same way a man or woman may have been pointed out as the embodiment of splendid health, and in all respects a magnificent type of humanity. Suddenly an accident happens, and in a moment, like the yacht, the perfect human creature falls crippled and helpless. In such an instance we have a case where the hygiene of illness may be studied with advantage. The shock of the accident is keenly felt, and often as keenly resented. The life of the patient may hang in the balance, the injuries may prove permanent in character, and yet the victim may benefit much. The enforced idleness, the endurance of suffering, the restraints of sickness, and the prolonged rest, may build up and restore parts of the organism which were becoming over-strained, and so extend life and promote powers of useful work. In this way the hygienic influence of illness becomes a reality, and its value cannot be questioned.

"The devil was sick, the devil a monk would be; the devil got well, the devil a monk was he." So, too, hale people who have never known a day's illness in their lives, often consider good health as an added virtue in their own case. The less fortunate who suffer from illness are regarded as foolish or stupid or at least as in some way lacking virtue. Good health may breed intolerance, inconsiderateness, or a selfish self-glorification. Now, it is a good thing to be healthy, but illness may bring blessings in its train. To be really ill is to produce thoughtfulness in the most ordinary of mortals. With thought comes a fairer sense of the proportion which health bears to individual merit. It teaches the value of kindness and skilful nursing, the power of ministering to others, the curse of selfishness, and the joy of doing something to leave the world a little better than we found it. There is then the mental and moral hygiene of illness which can do more to prevent the suicidal glorification of selfish souls than ordinary hygiene can do for people's bodies. Let us, therefore, give illness its due place. It may prove in its course unpleasant, painful, interfering, and hard to put up with, but against all this illness, when accepted in the proper spirit, cannot fail to water barren soil, to destroy the spirit of self, to open the eyes to a higher and nobler philosophy, and to cut the patient off for ever from paths which lead to destruction. So illness rightly understood should in most, if not in every instance, prove a cause for thankfulness. There is also the joy of illness. Who that has ever been a convalescent, that is to say, a patient who, after much suffering, has reached the stage where disease has ceased and health has to be restored, and will not heartily admit the joys of illness? What more happy state can life provide than one where the invalid becomes conscious once more of the beauties of Nature, of the flowers, the sunshine, the fresh air, and the busy hum of life. Each day as strength increases and illness retreats into the background we become more and more joyous. Friends are once more beside us, books can again charm, every day brings more and more pleasures of life within our reach, and so promotes a feeling of joy altogether innocent, and yet the completest of all the joys we have ever experienced.

There is a further joy, too! We can see as we write a dear friend, long since dead, who was brought by illness out of the darkness and misery of absolute worldliness into the glorious fulness of the joy of heaven

For months, the dire suffering and agony of that illness were resented with bitter repining, but gradually the good seed took root, and the latter weeks were marked by a joyous recognition of the blessings which God in his mercy had brought to the sufferer through the dark shadows of a terrible illness. Yes, illness may prove a fruitful source of joy. The man or woman who leaves selfishness behind, when recovery takes place, the thoughtless person who re-enters the world of health as a man with a mind, and the poor sufferer who dies happy because a merciful Providence has conducted him out of the world along the pathway of sickness, are all proofs of the benefits which illness may bring in its train. We hope that what we have written, incomplete or imperfect though it be, may bring comfort and conviction to some minds, at any rate, who have heretofore been taught to regard illness as an unmixed evil, instead of a blessing in disguise.

